# Dietary and Behavioral Interventions Protect against Age Related Activation of Caspase Cascades in the Canine Brain

**DOI:** 10.1371/journal.pone.0024652

**Published:** 2011-09-13

**Authors:** Shikha Snigdha, Nicole Berchtold, Giuseppe Astarita, Tommy Saing, Daniele Piomelli, Carl W. Cotman

**Affiliations:** 1 Institute for Memory Impairments and Neurological Disorders, University of California Irvine, Irvine, California, United States of America; 2 Department of Pharmacology, University of California Irvine, Irvine, California, United States of America; 3 Unit of Drug Discovery and Development, Italian Institute of Technology, Genoa, Italy; National Institute on Aging Intramural Research Program, United States of America

## Abstract

Lifestyle interventions such as diet, exercise, and cognitive training represent a quietly emerging revolution in the modern approach to counteracting age-related declines in brain health. Previous studies in our laboratory have shown that long-term dietary supplementation with antioxidants and mitochondrial cofactors (AOX) or behavioral enrichment with social, cognitive, and exercise components (ENR), can effectively improve cognitive performance and reduce brain pathology of aged canines, including oxidative damage and Aβ accumulation. In this study, we build on and extend our previous findings by investigating if the interventions reduce caspase activation and ceramide accumulation in the aged frontal cortex, since caspase activation and ceramide accumulation are common convergence points for oxidative damage and Aβ, among other factors associated with the aged and AD brain. Aged beagles were placed into one of four treatment groups: CON – control environment/control diet, AOX– control environment/antioxidant diet, ENR – enriched environment/control diet, AOX/ENR– enriched environment/antioxidant diet for 2.8 years. Following behavioral testing, brains were removed and frontal cortices were analyzed to monitor levels of active caspase 3, active caspase 9 and their respective cleavage products such as tau and semaphorin7a, and ceramides. Our results show that levels of activated caspase-3 were reduced by ENR and AOX interventions with the largest reduction occurring with combined AOX/ENR group. Further, reductions in caspase-3 correlated with reduced errors in a reversal learning task, which depends on frontal cortex function. In addition, animals treated with an AOX arm showed reduced numbers of cells expressing active caspase 9 or its cleavage product semaphorin 7A, while ENR (but not AOX) reduced ceramide levels. Overall, these data demonstrate that lifestyle interventions curtail activation of pro-degenerative pathways to improve cellular health and are the first to show that lifestyle interventions can regulate caspase pathways in a higher animal model of aging.

## Introduction

Aging is characterized by cognitive decline, synaptic dysfunction, and the accumulation of brain pathology. Lifestyle interventions such as diet, exercise, and cognitive training have emerged as effective strategies to prevent cognitive decline and reduce brain pathology [1], [2], [3]. However, how these lifestyle interventions improve the health of the aging brain remains unclear.

For several years now, our group has used the aged canine to study the specific effects of lifestyle interventions on brain aging and cognitive function. Like aged humans, the aged canine brain naturally shows cognitive decline, increased oxidative damage, mitochondrial dysfunction, selective neuron loss, and beta-amyloid (Aβ) accumulation [4]. We have demonstrated that long-term (2.8 years) behavioral enrichment (with social, cognitive, and exercise stimulation), dietary supplementation (with antioxidants and mitochondrial cofactors), or the combination of the behavioral and dietary interventions slow age-related cognitive decline and reduce brain pathology in aged canines [4], [5], [6]. The combination treatment was particular effective in improving cognitive performance [4], suggesting that the effects of the behavioral and dietary treatment approaches are additive.

Mitochondrial dysfunction, oxidative damage and Aβ accumulation are thought to be primary factors contributing to declining function in aging and Alzheimer's disease (AD) [7]. We have evaluated these mechanisms as potential targets of the behavioral and dietary interventions, and foundprominent effects on improving mitochondrial function and reducing oxidative stress. Notably, the interventions improved mitochondrial NADH respiration, reduced generation of mitochondrial reactive oxygen species (ROS) [8] lowered levels of protein carbonyls, and bolstered antioxidant defense mechanisms in the brain [9]. The reduction in oxidative damage correlated with cognitive improvement [9], suggesting that accumulating oxidative stress is likely to be a central feature underlying cognitive decline in aging. In parallel with reducing oxidative damage, the behavioral and dietary interventions modestly reduced the accumulation of Aβ in the aged canine brain, particularly with the combined intervention [10]. Surprisingly, while Aβ load was reduced in entorhinal, cingulate and parietal cortices, improvements in cognitive performance did not correlate with Aβ load [10], suggesting that effects on Aβ are likely not a central mechanism underlying the cognitive benefits of the interventions. However, because Aβ is well established to compromise neuronal health and synaptic function [11], [12], accumulation of Aβ in the aged canine brain is likely not benign, and it must be considered that AB may trigger downstream mechanisms that contribute to declines in brain health and cognitive function with age.

It is likely that the combined effects of oxidative stress, impaired mitochondrial function and AB accumulation can propagate harmful cascades that converge on common downstream mechanisms that ultimately cause neuronal damage and dysfunction. Two downstream targets that have recently come into increasing focus for their roles in compromising synaptic function and cognition are activated caspase 3 and the bioactive lipid ceramide. While caspases are best known for their role in apoptosis, recent evidence implicates caspase 3 in non-apoptotic processes, including impairing synaptic plasticity [13], spine atrophy and degeneration [14], [15], and memory deficits [14] in the absence of neuron loss. In addition, caspase 3 mediates some of the harmful effects of certain pathologies such as Aβ, as inhibition of long term potentiation (LTP) by Aβ1–42 is dependent on caspase 3 activation [13]. Like caspase 3, ceramides have recently been identified as potential causes of cognitive decline and onset of AD. Ceramide levels are elevated in the brain even at the earliest clinical stages of AD [16], and there is evidence that ceramide in CSF and serum may be a useful biomarker predicting cognitive decline [17]. These signaling molecules are important in an array of physiological processes and are generated in response to inflammatory cytokines and oxidative stress either by hydrolysis of sphingomyelin or by de novo synthesis [18], [19]. One effect of ceramide is to suppress the mitochondrial respiratory chain, resulting in increased production of ROS and oxidative stress [20]. Oxidative stress, in turn, can activate caspase 3 [21], [22], [23], and stimulate ceramide generation [24] revealing that oxidative stress, ceramides, and caspase 3 are linked in a self-perpetuating feed-forward cycle. Taken together, this literature suggests that oxidative damage, mitochondrial dysfunction and AB might ultimately converge on caspase 3 to impair synaptic and cognitive function.

In this study, we build on our previous findings that long-term behavioral enrichment, dietary supplementation, or the combined therapies improve cognitive function, improve mitochondrial health and function, reduce oxidative damage, and decrease AB in the aged canine brain. Using this same set of dog tissue, here we investigate if caspase activation and ceramide accumulation are reduced in the aged frontal cortex, serving as potential readout targets of intervention efficacy. In particular, we focus on caspase 3, based on its recently identified role in driving synaptic dysfunction in mouse models of AD. We assess if dietary, behavioral, or the combined interventions reduce the extent of caspase 3 activation in the aged canine frontal cortex, and assess potential roles of caspases 8 and 9 in mediating the effects of the interventions on caspase 3. In parallel, we assess levels of several species of ceramides, candidate risk factors for triggering pathological cascades and cognitive impairment in the aging brain.

## Results

### Activated caspase 3 is reduced with ENR, AOX, or combined intervention

**Figure 1:**
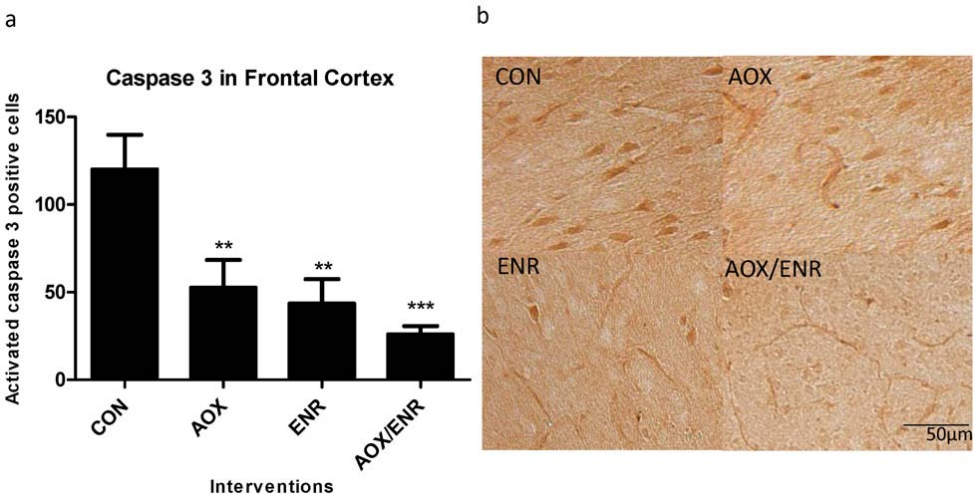
Immunohistochemical staining for caspase 3 in aged canine brains. **a**) Caspase 3 immunohistochemical staining in frontal cortices of aged dogs treated with AOX and/or ENR interventions showed significant reduction in expression of active caspase 3. **p<0.01,***p<0.001. CON: control environment/control diet; AOX: control environment/antioxidant diet; ENR: behavioral enrichment/control diet; AOX/ENR: behavioral enrichment/antioxidant diet **b**) representative images of caspase 3 staining in frontal cortices.

**Figure 2:**
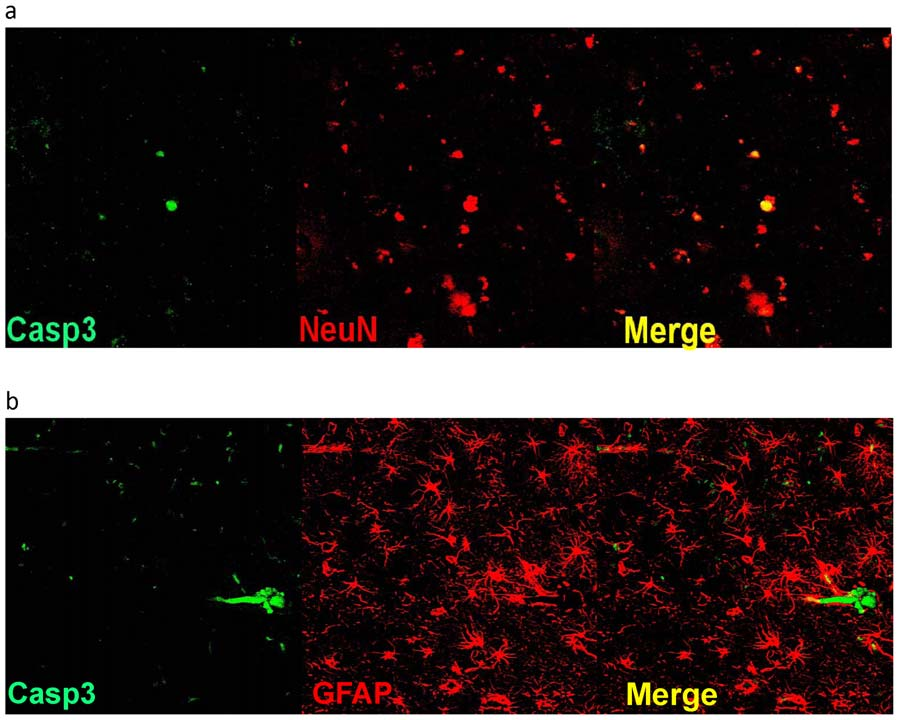
Double labeling with caspase 3 and neuronal or glial markers. **a**) Double labeling of activated caspase 3 with the neuronal marker NeuN and **b**) the glial marker GFAP revealed that the majority of cells positive for activated caspase-3 were also NeuN positive.

To evaluate if behavioral enrichment (ENR), dietary enrichment (AOX), or the combined intervention (ENR/AOX) affected the extent of caspase 3 activation in the frontal cortex, cells immune-positive for activated caspase 3 were counted. An antibody specific to activated caspase 3 was used that detects the large (17 kDa) fragment but that does not recognize either the full-length procaspase-3 or other cleaved caspases. One-way ANOVA identified a significant intervention effect (F_3,20_ = 8.3; p<0.01) and post-hoc analysis revealed fewer cells positive for activated caspase 3 following AOX (p<0.01), ENR (p<0.01) and ENR/AOX (p<0.001) treatment relative to aged controls. The greatest reduction occurred with the combined treatment, which reduced counts of activated caspase-3 positive cells by 80% relative to untreated control values (Fig. 1). Double labeling of activated caspase 3 with the neuronal marker NeuN (Fig. 2a) and the glial marker GFAP (Fig. 2b) revealed that the majority of cells positive for activated caspase-3 were also NeuN positive. These data demonstrate that activated caspase 3 is present in the aged dog frontal cortex, particularly in neurons, and that ENR and AOX interventions potently reduce the number of cells in which this potentially harmful protease is activated.

### Lower levels of activated caspase 3 activity correlate with better discrimination learning

We next evaluated if the extent of caspase 3 activation related to cognitive performance, with the prediction that better cognition would be present in those animals where fewer cells contained activated caspase 3. To determine whether caspase activation was correlated with cognitive function, we used behavioral data from our previously published findings [5].

**Figure 3:**
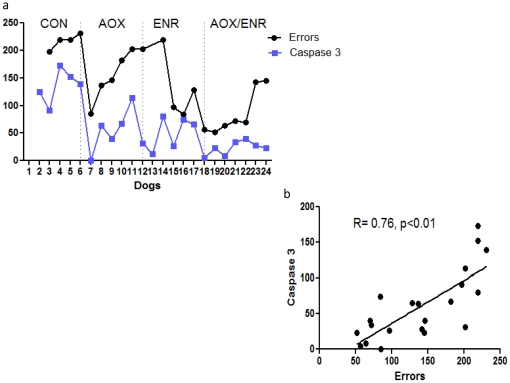
Lower levels of activated caspase 3 activity correlate with better discrimination learning. **a**) Parallel relationship between cognitive scores (errors in reversal learning) and abundance of cells expressing activated caspase 3 in each animal **b**) Caspase 3 expression is directly correlated with errors in reversal learning. Pearson analysis r = 0.76, p<0.01.

We selected a task that engages frontal lobe function (black/white discrimination learning and reversal) and that was conducted at the end of the 2.8 yr intervention period [5], so that the molecular state would correspond closely to that present at the time of testing. Plotting cognitive scores (errors in reversal learning) and abundance of cells expressing activated caspase 3 revealed a strikingly parallel relationship between these 2 variables in each animal (fig. 3a). Indeed, Pearson analysis identified a robust correlation (r = 0.76, p<0.01) across the 24 dogs (control, ENR, AOX, ENR/AOX) (Fig. 3b). These results suggest that beneficial effects of the interventions on cognitive function may be related to reduced abundance of activated caspase 3.

### Activated caspase 3 is associated with increased levels of caspase-3 cleavage products

We next evaluated levels of casapase-3 cleavage products to confirm that reduced immunoreactivity for activated caspase 3 with the interventions also reduced downstream proteolytic consequences. Two well-characterized cleavage targets of caspase 3 are the cytoskeletal proteins beta-actin and tau. Specifically, activated caspase-3 cleaves beta-actin between Asp244 and Gly245, generating a 32 kDa N-terminal fragment of actin known as Fractin, while tau is cleaved at the D421 site (tau_421_). Using an activity test followed by dot-blot analysis, we confirmed that caspase 3 cleaves tau to produce tau_421_ in canine frontal cortex homogenates (data not shown), similar to the effect of caspase 3 on tau in human and rodent brain tissue.

**Figure 4:**
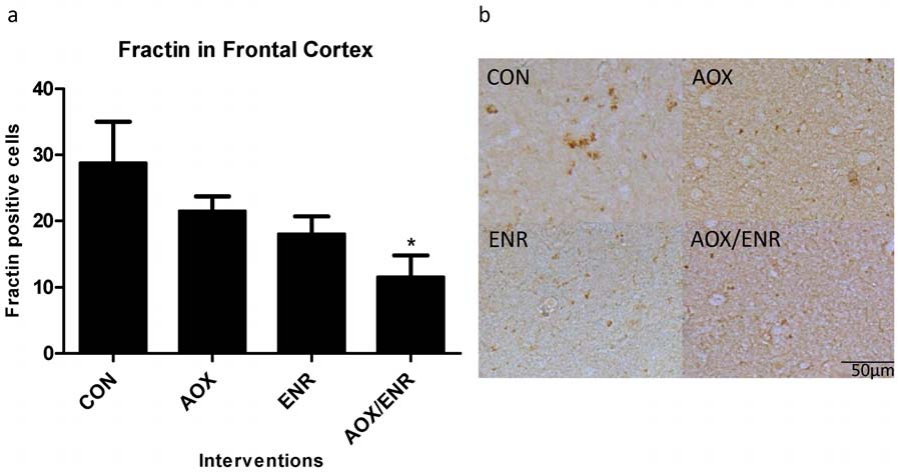
Activation of caspase 3 is associated with increased levels of caspase-3 cleavage products. **a**) Fractin immunohistochemical staining in frontal cortices of aged dogs treated with AOX and/or ENR interventions showed significant reduction in expression of active caspase 3 in the E/A group *p<0.05. CON: control environment/control diet; AOX: control environment/antioxidant diet; ENR: behavioral enrichment/control diet; AOX/ENR: behavioral enrichment/antioxidant diet **b**) representative images of fractin staining in frontal cortices.

**Figure 5:**
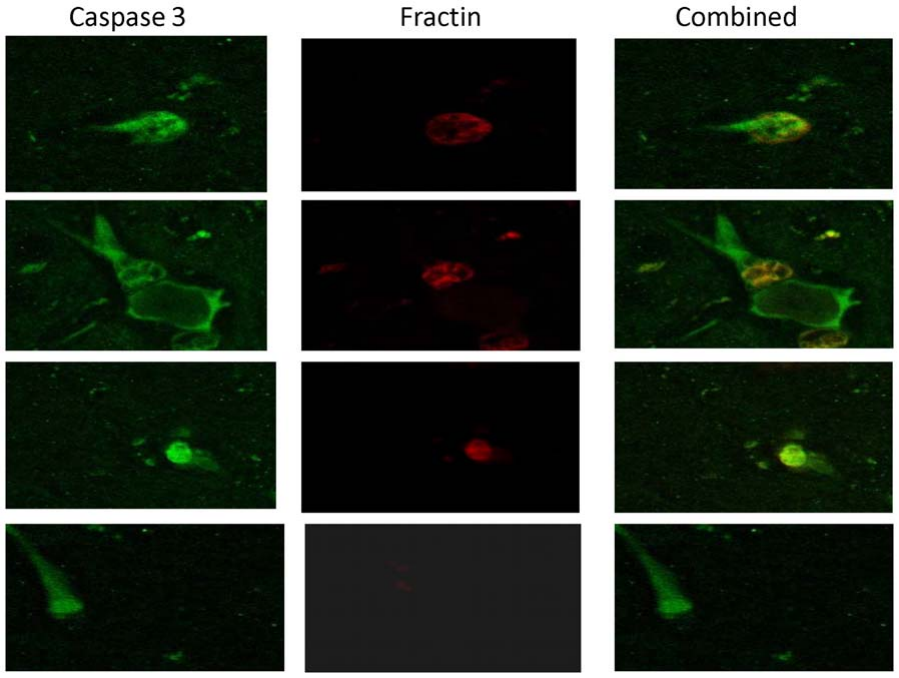
Double-labeling detected by immunofluorescence revealed colocalization of fractin and active caspase 3 in several cells in the frontal cortex.

Frontal cortex sections were immunolabeled with an antibody specific to fractin and cell counts were assessed in CON, AOX, ENR and AOX/ENR groups. One-way ANOVA revealed a significant treatment effect (F_3,20_ = 3.30, p<0.05), with modest reductions in fractin-positive cells in the AOX and ENR groups and a significant reduction to 40% of untreated controls in the ENR/AOX combined treatment group (p<0.05) (Figs. 4a, b). Double-labeling experiments revealed colocalization of fractin and active caspase 3 in several cells in the frontal cortex (Fig. 5). Dot blot assays were used to assess if levels of tau_421_ were similarly affected by the interventions. One-way ANOVA revealed a significant main effect (F_3,20_ = 3.52, p<0.05), with tau_421_ levels reduced by 30% relative to untreated control levels with AOX (p<0.05) and AOX/ENR intervention (p<0.05), and a non-significant 22% decrease following ENR alone (data not shown). In addition, Pearson correlation revealed that abundance of active caspase 3 correlated with levels of both tau_421_ (r = .53, p<0.01), and fractin (r = 0.7, p<0.001). Taken together, these data indicate that activation of caspase 3 and subsequent cytoskeletal protein degradation are reduced in the frontal cortex of aged dogs following AOX and ENR interventions, with the most robust effect in the combined ENR/AOX treatment group.

### Activation of caspase 9, but not caspase 8, is reduced by the interventions

**Figure 6:**
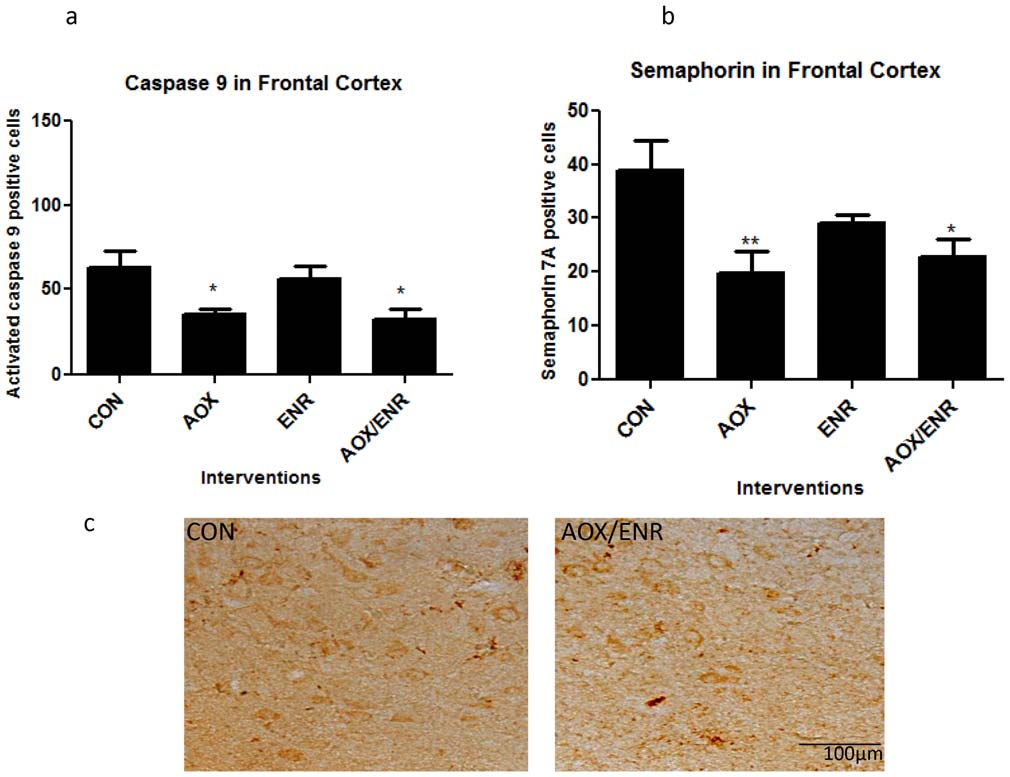
Interventions reduce activation of caspase 9 and semaphorin 7a expression in aged canine brains. **a**) Active caspase 9 immunohistochemical staining in frontal cortices of aged dogs treated with AOX and/or ENR interventions showed significant reduction in expression of active caspase 9 in the AOX and combined treatment groups *p<0.05. **b**) Expression of semaphorin 7a, was reduced in frontal cortices of aged dogs in the AOX (**p<0.01) and AOX/ENR (p<0.05) groups. **c**) Representative images of semaphorin 7a staining in frontal cortices of CON and AOX/ENR groups.

Because caspase 3 is primarily activated by two initiator pathways driven by caspases 8 and 9, we next investigated if either of these caspases may be a mechanism by which the dietary and behavioral interventions reduce caspase 3 activation. Immunohistochemistry using an antibody specific to activated caspase 8 revealed the presence of this activated caspase in frontal cortical sections, however the incidence of immune-positive cells was rare. Activated caspase-3 was detected in all groups, suggesting that the effects of the dietary and behavioral interventions on caspase 3 activation are not mediated via caspase 8. On the other hand, the interventions reduced caspase 9 activation, based on counts of cells immunopositive for active caspase 9 and semaphorin 7A, a specific cleavage product of active caspase 9. One-way ANOVA revealed a significant treatment effect on cells positive for active caspase 9 (F_3,18_ = 4.85, p = 0.016, Fig. 6a). Relative to untreated aged controls, activated caspase 9 positive cell counts were reduced by 45% with AOX (p<0.05) and 50% with AOX/ENR (p<0.05) intervention, with a non-significant 19% decrease with ENR alone. Further, one-way ANOVA of semaphorin-7a positive cell counts revealed a significant treatment effect of the interventions on the cleavage product of caspase 9 (F_3,20_ = 4.98, p = 0.013). Relative to untreated aged controls, semaphorin 7A positive cell counts were reduced 50% following AOX (p = 0.017) and 42% with the combined AOX/ENR (p<0.05) intervention, with a smaller (25%) non-significant decrease with ENR alone (Fig. 6b, c). Overall, these data indicate that caspase 9 activity was reduced with interventions containing an AOX arm, with little additional effect from the behavioral intervention, and suggest that reductions in activation of caspase 9, but not caspase 8, may account in part for decreased caspase 3 activation with the dietary intervention.

### Effect of ENR and AOX on ceramide mobilization

**Figure 7:**
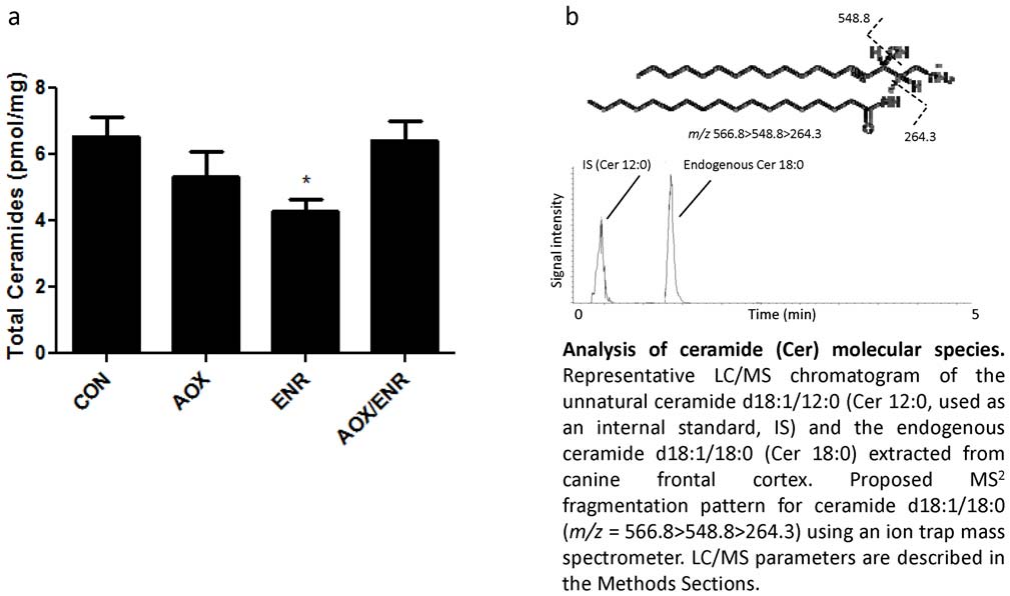
Effect of ENR and AOX on ceramide mobilization. **a**) Levels of total ceramides in the frontal cortices of aged dogs treated with AOX and/or ENR interventions showed significant reduction in ENR group only (p<0.05). **b**) Representative LC/MS chromatogram of the unnatural ceramide d18:1/12:0 (Cer 12:0, used as an internal standard, IS) and the endogenous ceramide d18:1/18:0 (Cer 18:0) extracted from canine frontal cortex.

The sphingolipid ceramide can disrupt the mitochondrial respiratory chain, activate caspases and has been implicated in signaling pathways that impair learning and memory. In particular, increases in the levels of ceramide species in brain and plasma were linked to cognitive impairment/decline in AD/MCI [17], [25]. To determine the effect of our interventions on ceramide levels, we assessed levels of several species of ceramides (14:0, 16:0, 18:0, 24:1) in the aged dog brain. The ceramide species d18:1/18:0 and d18:1/14:0 showed treatment effects (F_3, 20_ = 3.08, p<0.05, Fig. 7a), with the strongest effect occurring with ENR alone. For the 18:0 species (F_3, 20_ = 3.08, p<0.05, Fig. 7), ENR reduced levels to 61% of untreated control levels (p<0.05). Similarly, the significant treatment effect on the 14:0 species was due to decreased levels with ENR treatment to 63% of untreated control levels (p = 0.014). No significant treatment effect was found for the other ceramide species (Table 1). Unexpectedly, there was no effect of AOX alone or combined AOX/ENR treatment on any ceramide species. These results suggest that AOX intervention does not affect ceramide generation, while ENR induces specific changes in cellular ceramide levels.

**Table 1:** Table showing levels of individual ceramide species as measured by LC/MS in the frontal cortices of aged dogs treated with dietary or behavioral interventions.

	CON	AOX	ENR	AOX/ENR
**Cer d18:1/14:0**	0.06±0.001	0.006±0.002	0.004±0.001	0.010±0.005
**Cer d18:1/16:0**	0.20±0.02	0.24±0.07	0.170±0.02	0.196±0.02
**Cer d18:1/18:0**	6.05±0.55	5.00±0.90	3.701±0.39	5.951±0.53
**Cer d18:1/24:1**	0.12±0.02	0.14±0.03	0.126±0.02	0.129±0.19

Data expressed as ±SEM. CON: control environment/control diet; AOX: control environment/antioxidant diet; ENR: behavioral enrichment/control diet; AOX/ENR: behavioral enrichment/antioxidant diet.

### Caspase 3 activation is not accompanied by apoptosis: TUNEL labeling

While caspase activation has traditionally been associated with apoptosis, recent findings reveal that activated caspase 3 can be present in the brain with no evidence of cell death. To determine if caspase 3 activation in the aged dog frontal cortex is accompanied by apoptosis, we used TUNEL-labeling to detect the DNA fragmentation characteristic of apoptotic cells. Interestingly, while extensive TUNEL-positive labeling was apparent in the positive control tissue (from a patient who had died from AD), no TUNEL-positive cells were identified in any of the four treatment groups of aged dogs, suggesting that activation of caspase 3 is not sufficient to trigger cell death in the aged canine brain.

## Discussion

Behavioral lifestyle interventions represent a quietly emerging revolution in the modern approach to counteracting age-related declines in brain health and cognitive function. In this study, we investigated if activation of caspases and ceramide accumulation are reduced by long term dietary and behavioral interventions. Caspase activation and ceramide accumulation are common convergence points for oxidative damage and AB, among other factors associated with the aged and AD brain. While activated caspases and ceramides are present in the AD brain and are known risk factors for cognitive decline and neurodegeneration, no studies to date have assessed if these are targeted by ENR, AOX or combined intervention using higher animal models of human brain aging.

Our data reveal a high abundance of cells with activated caspase 3 in the frontal cortex of the aged dog brain, present primarily in neurons. In parallel, caspase cleavage products, such as cleaved cytoskeletal proteins actin and tau are present. A sustained change in lifestyle consisting of behavioral enrichment, dietary supplementation with antioxidants and mitochondrial cofactors, or the combined intervention dramatically reduced the abundance of cells expressing activated caspase 3, with the combined intervention having the greatest effect, reducing numbers of immune-positive cells by 80% relative to untreated levels. The reduction in active caspase 3 correlated strongly with reduced error scores on discrimination learning, suggesting that reduction in caspase 3 activation is an important target of the interventions to promote cognitive stability with age. In parallel with fewer cells expressing active caspase-3, there were fewer cells expressing caspase cleavage products, including fractin and cleaved tau, which may underlie the benefits of reduced caspase 3 activation. For example, the truncation state of tau influences many of its normal and pathologic characteristics, including its ability to bind to and stabilize microtubules [26], [27] and thereby may compromise axonal flow and intracellular trafficking. Taken together, these data suggest that reduced caspase 3 activation is likely to be an important mechanism underlying benefits of the interventions on maintaining cognitive function with age.

Caspase 3 is traditionally known as one of the primary effector caspases driving apoptosis. However, our finding that activated caspase 3 is not accompanied by TUNEL labeling strongly suggests that caspase activation in the canine brain is associated with non-apoptotic processes. This is supported by our previous finding that neuron loss in the aged canine brain is minimal [28], consistent with the general absence of cortical neuronal loss in normal human brain aging [29]. Further, reports of active caspase 3 in absence of apoptosis have recently emerged. For example, in olfactory sensory neurons, caspases 3 and 9 are activated without cells undergoing histone H1 changes or apoptotic morphology [30]. Similarly, activated caspases (including caspase 3) are present in cortical neurons of transgenic mouse models of AD in the absence of cell death [31]. These findings indicate that activated caspase 3 can be dissociated from apoptosis, and may be linked to other cellular processes. Indeed, recent findings demonstrate that activate caspase 3 is a key factor impairing synaptic function. For example, an up-regulation of caspase 3 mediates the inhibition of long term potentiation (LTP) by Aβ1–42 [13] and synaptic dysfunction [14]. Taken together, these findings suggest that reducing caspase 3 activation is beneficial to synaptic health and neuronal function, and may be an important mechanism targeted by the dietary and behavioral interventions to improve cognitive function.

While activation of caspase 3 is regulated primarily by the initiator caspases 8 and 9, our data suggest that only caspase 9 activation is targeted by the interventions. Active caspase 8 was present but sparse in the aged frontal cortex, and was detected in all treatment groups. In contrast, many cells were immunopositive for active caspase 9, with animals treated with an AOX arm (eg AOX, or AOX/ENR) showing approximately 50% fewer cells expressing active caspase 9 or its cleavage product semaphorin 7A. Because caspase 9 is selectively released by stressed mitochondria these findings suggest that mitochondrial health is improved by the interventions, consistent with our previous finding that the antioxidant diet reduced mitochondrial ROS production and improved NADH respiration in the canine [32].

Like caspase 3, the bioactive lipid group of ceramides has recently been identified as potential causes of cognitive decline and onset of AD. Levels of ceramides have been shown to be elevated in the cerebral cortex during normal aging and in Alzheimer's disease [33]. It has been hypothesized that the harmful effects of ceramide are caused by suppression of the mitochondrial respiratory chain, with resulting ROS production and oxidative stress [20]. Because our previous data demonstrated that the AOX diet reduced mitochondrial ROS production and improved NADH respiration in the canine [32]we hypothesized that a reduction in ceramide might be one mechanism targeted by the dietary intervention. Unexpectedly, the AOX diet, either alone or combined with ENR, did not reduce ceramide levels in the aged cortex, contrasting with the pronounced declines in caspase 3 activation with the combined intervention. These data indicate that the reduction in caspase 3 activation with the interventions is not tightly linked to ceramide levels in the aged dog brain. Finally, the finding that ceramide levels were reduced with behavioral enrichment alone suggests that behavioral intervention can improve cellular health by pathway(s) distinct from those engaged by the dietary interventions. Ceramide reduction with behavioral enrichment may contribute to the cognitive benefits derived from ENR alone.

Taken together, our findings reveal that life style interventions can engage a range of molecular mechanisms to improve brain health and cognitive function. Along with our previous findings that behavioral and dietary interventions improve mitochondrial NADH respiration, reduce generation of mitochondrial reactive oxygen species (ROS) [8] lower levels of protein carbonyls, and bolster antioxidant defense mechanisms in the brain [9] and increase levels of brain-derived neurotrophic factor (BDNF), [34], we demonstrate here that these interventions attenuate caspase 3 activation in the aged frontal cortex. In addition to decreasing harmful factors that accumulate with age and impair neuronal health and function, lifestyle interventions increase factors that are protective and promote plasticity. Since many of these mechanisms decrease caspase 3 activation [35], [36] these data support the idea that regulation of caspase 3 activation may be an important convergence point for multiple pathways activated by lifestyle interventions. Overall, these data are the first to show that lifestyle interventions can regulate caspase pathways in a higher animal model of human brain aging.

## Materials and Methods

### Subjects

Twenty-four beagles ranging in age at the start of the study from 8.05 to 12.35 years (mean = 10.69 years, SE = 0.25, 12 males/12 females) were obtained from the colony at the Lovelace Respiratory Research Institute. Animals were born and maintained in the same environment and all had documented dates of birth and comprehensive medical histories.

### Ethics Statement

All studies were conducted in compliance with approved IACUC protocols, consistent with the National Research Council's Guide for the care and use of laboratory animals.

### Group assignments and study timeline

All dogs underwent extensive baseline cognitive testing as has been described previously [32], [37]. Based on cognitive test scores, animals were ranked in order of cognitive ability and placed into one of four treatment groups such that each group contained animals with equivalent ranges of cognition (e.g. poor to good): control environment/control diet (CON), enriched environment/control diet (ENR), control environment/antioxidant diet (AOX), enriched environment/antioxidant diet (ENR/AOX).

### Behavioral enrichment

The behavioral enrichment protocol has been described previously [32] and consisted of housing animals in pairs (social enrichment), providing 2–20 min outdoor walks per week (physical exercise), and continuous cognitive testing (cognitive enrichment). The cognitive enrichment consisted of a landmark discrimination task [38], an oddity discrimination task [39], and a size discrimination learning and reversal task [5], [40].

### Diet

Both the control and supplemented test foods were formulated to meet the nutrient profile for the American Association of Feed Control Officials (AAFCO) recommendations for adult dogs (AAFCO 1999) and has been described previously [32]. Control and test foods were identical in composition, with the exception that the test diet contained a broad-based antioxidant and mitochondrial cofactor supplementation. The control and enriched foods had the following differences in formulation on an as-fed basis, respectively: dl-alpha-tocopherol acetate, (120 ppm vs 1050 ppm), l-carnitine (<20 ppm vs 260 ppm), dl-alpha-lipoic acid (<20 ppm vs 128 ppm), ascorbic acid as Stay-C (<30 ppm vs 80 ppm), and 1% inclusions of each of the following (1-to-1 exchange for corn): spinach flakes, tomato pomace, grape pomace, carrot granules, and citrus pulp.

### Tissue Preparation

Dogs were ex-sanguinated under anesthesia (5% isoflurane), by cardiac puncture and within 15 min the brain was removed from the skull. The brain was sectioned midsagitally, with the entire left hemisphere being immediately placed in 4% paraformaldehyde for 48–72 h at 4°C then transferred to phosphate buffered saline with 0.05% sodium azide at 4°C for long term storage. The left hemisphere was sent to NeuroScience Associates for sectioning. NeuroScience Associates treated individual canine hemispheres with 20% glycerol and 2% dimethylsulfoxide to prevent freeze-artifacts and subsequently embedded two hemispheres (i.e. two animals) per block in a gelatin matrix using MultiBrain Technology™ (NeuroScience Associates, Knoxville, TN; http://www.neuroscienceassociates.com/multibrain.htm). The MultiBrain™ block was sectioned coronally at 40 µm. All sections cut (none were discarded) were collected sequentially into a 4×6 array of containers which were filled with Antigen Preserve solution (50% PBS pH 7.0, 50% ethylene glycol, 1% polyvinyl pyrrolidone) for sections to be immunohistochemically stained. At the completion of sectioning, each container held a serial set of one-of-every-24th section (or one section every 960 µm), with approximately 1400 sections generated per hemisphere. One container of free-floating sections was randomly selected per dog and all sections in that container that frontal cortex were collected for immunohistochemical staining.

### Immunohistochemistry

Standard immunohistochemical methods were used and have been published elsewhere [40], [41]. The following primary antibodies were used: active caspase 3 (1∶100; chemicon), active caspase 8 (1∶200), active caspase 9 (1∶100; abcam), semaphorin 7A (1∶100; abcam), caspase-cleaved tau (1∶1000; generously supplied by L Binder), fractin (1∶500; chemicon), xIAP (1∶200;Abcam). Bound secondary biotinylated IgG (Vector Laboratories, Burlingame, CA) was detected using ABC peroxidase kits from Vector Laboratories (Burlingame, CA). Free-floating sections were mounted onto gelatin coated slides, dehydrated through ethanols and coverslipped with DePeX mounting medium. One section from each of the 24 dogs was stained in each immunohistochemistry run. Control experiments where primary or secondary antibody was omitted resulted in negative staining. Complete penetration of the antibodies was confirmed by detection of labeled cells in all deeper layers within a section. For quantification of staining, total numbers of labeled cells were counted in at least three different sites from each section, using a light microscope at 20× magnification.

### Immunofluorescent double labeling

Immunofluorescence was used to evaluate colocalization of cleaved caspase 3 (1∶100) with either NeuN (1∶500) or GFAP (1∶1000). The primary antibody/antigen complex was detected using secondary antibodies conjugated to either Alexa 488 or Alexa 568 (Invitrogen), and was visualized using a Zeiss LSM510 META confocal system configured with a Zeiss Axiovert 200 M motorized inverted microscope. Multilabeled fluorescent samples were imaged acquiring each fluorescent channel sequentially to avoid signal bleed-over. The Alexa 488 component was obtained using the 488 nm line of an argon ion laser for excitation and a band pass 505–530 nm emission filter. The Alexa 564 component was obtained using the 543 nm line of a green helium/neon laser for excitation and a band pass 585–620 nm emission filter.

### Dot blot assay

Nitrocellulose membranes were dotted with 1 µl of frontal cortical crude extracts (4 ug/ul) from 4 aged beagles. All extracts were suspended in 100 µl of Laemmli buffer prior to absorption on the membrane. The membrane was air dried for 30 minutes and blocked with TBS-5% BSA and incubated with caspase cleaved tau antibody (1∶1000, overnight at 4°C). After three washes with TBS-0.05% Tween 20, the immune complexes were revealed with HRP-labeled goat anti-mouse secondary antibody (Sigma) (1/10,000 dilution, 1 hour at room temperature) followed by ECL detection. In addition, to detect caspase 3-induced cleavage of tau in crude frontal cortex homogenates, an activity test was performed. Specifically, wells were coated with 1 µl of active caspase-3 protease (Chemicon) and incubated with frontal cortex homogenates (1 µl, 4 ug/ul) from 4 aged beagles at 37°C for 1 hour. A dot blot assay to detect cleaved tau was then performed on the samples as described above.

### Ceramide analyses

Lipid extractions analysis was conducted as previously described [42]. Briefly, frozen tissue samples were weighed and homogenized in cold methanol containing appropriate authentic standards (listed below). Total lipids were extracted by adding chloroform and water (2/1, vol/vol) and fractionated through open-bed silica gel columns by progressive elution with chloroform/methanol mixtures. Fractions eluted from the columns were dried under nitrogen, reconstituted in chloroform/methanol (1∶4, vol/vol; 0.1 ml) and subjected to liquid chromatography/mass spectrometry. Ceramides were analyzed by tandem mass spectrometry, using an Agilent 1100 liquid chromatograph coupled to an ESI-ion-trap XCT mass detector. Ceramide molecular species were separated on a Poroshell 300 SB C18 column (2.1×75 mm i.d., 5 µm; Agilent Technologies) maintained at 30°C. A linear gradient of methanol in water containing 5 mM ammonium acetate and 0.25% acetic acid (from 80% to 100% of methanol in 3 min) was applied at a flow rate of 1 ml/min. Detection was in the positive mode, capillary voltage was 4.5 kV, skim1 −40 V, and capillary exit −151 V. Nitrogen was used as drying gas at a flow rate of 12 L/min, temperature of 350°C, and nebulizer pressure of 80 psi. Helium was used as collision gas. Ceramide species were identified by comparison of its LC retention time and MSn fragmentation pattern with that of authentic standards (Avanti Polar Lipids). Extracted ion chromatograms were used to quantify the following ceramides: d18:0/14:0 [M+H]^+^ (m/z = 510.5>492.5>264.3), d18:1/16:0 [M+H]^+^ (m/z = 538.5>520.5>264.3), d18:1/18:0 [M+H]+ (m/z = 566.5>548.5>264.3), d18:1/24:1 [M+H]^+^ (m/z = 648.6>630.8>264.3), using d18:1/12:0 [M+H]^+^ (m/z = 482.5>464.5>264.3) as an internal standard.

### Terminal dUTP Nick-end Labeling and Staining

Apoptotic cells can be detected by terminal deoxynucleotidyl transferase (TdT)-mediated dUTP nick end labeling (TUNEL). Using slide-mounted tissue sections, DNA fragmentation was detected using a TUNEL labeling apoptosis detection kit (Chemicon) according to the manufacturer's instruction. As a positive control, a brain section from a patient who had died from AD was included.

### Statistical analysis

To quantify immunohistochemical staining, numbers of labeled cells were counted in at least three different sites from each frontal cortex section, using a light microscope at 20× magnification. For each section, values from the multiple sites were averaged to generate a single mean value per dog. Average and standard error of the mean (n = 5–6 per group) were then generated for each treatment group. Dot blots were quantified by using Image J software and the intensity of the dots are expressed as optical density (OD). Statistical analysis on immunopositive cell counts, dot blot intensity, and ceramide levels consisted of one-way ANOVA across the 4 treatment groups to detect a main effect of intervention, followed by post-hoc bonferroni t-test (p<0.05) if a significant main effect was detected.
